# Computational modelling of amino acid exchange and facilitated transport in placental membrane vesicles

**DOI:** 10.1016/j.jtbi.2014.10.042

**Published:** 2015-01-21

**Authors:** N. Panitchob, K.L. Widdows, I.P. Crocker, M.A. Hanson, E.D. Johnstone, C.P. Please, C.P. Sibley, J.D. Glazier, R.M. Lewis, B.G. Sengers

**Affiliations:** a Bioengineering Science Research Group, Faculty of Engineering and the Environment, University of Southampton, Southampton, UK; b Maternal & Fetal Health Research Centre, Institute of Human Development, University of Manchester, Manchester, UK; c St. Mary’s Hospital & Central Manchester University Hospitals NHS Foundation Trust, Manchester Academic Health Science Centre, Manchester, UK; d Faculty of Medicine, University of Southampton, Southampton, UK; e Mathematical Institute, Oxford University, Oxford, UK; f Institute for Life Sciences, University of Southampton, Southampton, UK

**Keywords:** Amino acids, Membrane transport, Antiport, Carrier model

## Abstract

Placental amino acid transport is required for fetal development and impaired transport has been associated with poor fetal growth. It is well known that placental amino acid transport is mediated by a broad array of specific membrane transporters with overlapping substrate specificity. However, it is not fully understood how these transporters function, both individually and as an integrated system. We propose that mathematical modelling could help in further elucidating the underlying mechanisms of how these transporters mediate placental amino acid transport.

The aim of this work is to model the sodium independent transport of serine, which has been assumed to follow an obligatory exchange mechanism. However, previous amino acid uptake experiments in human placental microvillous plasma membrane vesicles have persistently produced results that are seemingly incompatible with such a mechanism; i.e. transport has been observed under zero-trans conditions, in the absence of internal substrates inside the vesicles to drive exchange. This observation raises two alternative hypotheses; (i) either exchange is not fully obligatory, or (ii) exchange is indeed obligatory, but an unforeseen initial concentration of amino acid substrate is present within the vesicle which could drive exchange.

To investigate these possibilities, a mathematical model for tracer uptake was developed based on carrier mediated transport, which can represent either facilitated diffusion or obligatory exchange (also referred to as uniport and antiport mechanisms, respectively). In vitro measurements of serine uptake by placental microvillous membrane vesicles were carried out and the model applied to interpret the results based on the measured apparent Michaelis–Menten parameters *K*_*m*_ and *V*_max_. In addition, based on model predictions, a new time series experiment was implemented to distinguish the hypothesised transporter mechanisms. Analysis of the results indicated the presence of a facilitated transport component, while based on the model no evidence for substantial levels of endogenous amino acids within the vesicle was found.

## Highlights

• Initial rate and time course data for serine uptake in placental membrane vesicles.
• Integrated model analysisof facilitative diffusion vs obligatory exchange.
• Dependency apparent Michaelis–Menten constants on internal concentrations.
• Uptake in placental vesicles was consistent with a facilitative transport component.
• No effects of any internal endogenous substrate in vesicles were apparent.

## 1 Introduction

Amino acid transfer across the placenta is an important determinant of fetal growth (Jansson et al., 2006, Paolini et al., 2001, Sibley et al., 2010). Impaired fetal growth is associated with poor neonatal outcomes and in adult life with increased rates of chronic disease (Lewis et al., 2012). While currently no interventions are available for growth restricted fetuses in utero, it is known that transfer of amino acids and other nutrients across the placenta is decreased in affected pregnancies (Paolini et al., 2001) and that activity of certain amino acid transport mechanisms is impaired (Glazier et al., 1997, Jansson and Powell, 2006, Sibley et al., 1997). Hence, an improved mechanistic understanding of placental transport could potentially lead to the development of targeted treatments to either prevent or alleviate intrauterine growth restriction.

Transfer of amino acids from the maternal blood, across the placenta and into the fetal blood, is a complex process in which amino acids need to cross both the maternal facing microvillous plasma membrane (MVM) and the fetal facing basal plasma membrane (BM) of the placental syncytiotrophoblast (Cleal and Lewis, 2008, Cleal et al., 2011). Transport of amino acids is mediated by specific membrane transporter proteins. These include: (i) Accumulative transporters, which can transport amino acids against their gradient using secondary active transport driven by the sodium electrochemical gradient, thereby building up high concentrations in the syncytiotrophoblast (Philipps et al., 1978). (ii) Exchangers (antiporters), which transfer one amino acid from outside of the plasma membrane in exchange for an amino acid from inside the cytosol. Thus, exchangers play an important role in altering the composition of amino acids, but not the net amount of amino acid transferred across the placenta. (iii) Facilitated transporters, which enable facilitated diffusion of amino acids down the prevailing concentration gradient, from the placental syncytiotrophoblast into the fetal circulation, resulting in net transport.

Given this complexity, experiments using isolated plasma membrane vesicles prepared from human placental MVM or BM are commonly used to measure in vitro amino acid uptake, allowing for transporter activity to be studied under controlled conditions (Glazier and Sibley, 2006, Lewis et al., 2007). The current study will focus on the sodium-independent transport of serine, which can be primarily attributed to the transporter protein LAT2 (SLC7A8) (Lewis et al., 2007). LAT2 is believed to be an obligatory exchanger (Broer, 2008, Meier et al., 2002) although one study has reported a non-obligatory component (Segawa et al., 1999). Furthermore, in previous placental vesicle studies, sodium-independent serine uptake has been observed when amino acids were initially nominally absent inside the vesicle (zero-trans experiment) (Lewis et al., 2007). However, this is incompatible with the concept of obligatory exchange, which requires amino acid to be present on both sides of the membrane in order for exchange to occur. Therefore, this gives rise to two alternative hypotheses: (i) That sodium independent transport of serine may not be fully obligatory, or alternatively (ii), there is an initial level of endogenous amino acids present inside the vesicle, which could then enable obligatory exchange.

Mathematical modelling could potentially help to test these hypotheses (Lewis et al., 2013). Previous placental modelling studies have mainly focussed on blood flow, oxygen transfer, and solute transport by simple diffusion (Chernyavsky et al., 2010, Gill et al., 2011). Placental models including relationships for membrane transport have been applied to model transport of drugs (Staud et al., 2006) and glucose (Barta and Drugan, 2010), but few modelling studies have specifically addressed the issue of placental amino acid transport (Sengers et al., 2010). Kinetic models for carrier-mediated solute transport by membrane transporters in general have been studied extensively in the past (Friedman, 2008, Läuger, 1991, Stein and Lieb, 1986). In addition, more recent advances in computational analysis have allowed simulation of transporter function based on knowledge of the detailed molecular structure (Khalili-Araghi et al., 2009). Nonetheless, in biological experiments, the well-known Michaelis–Menten equation is most commonly applied to describe saturable transport processes (Jóźwik et al., 2004, Lewis et al., 2007, Meier et al., 2002). However, this equation does not fully represent many important transport phenomena, for instance facilitated diffusion and exchange transporters, which are intrinsically dependent on substrate concentrations on both sides of the plasma membrane. Thus, while this approach is useful to describe apparent transport properties under specific conditions (e.g. initial uptake rates), more complex mechanistic models are required to capture transporter behaviour under various physiological conditions.

The aim of this study was to use mathematical modelling to further elucidate the potential mechanisms of sodium-independent transport of serine in placental MVM vesicles. For this purpose, a standard vesicle experiment was carried out and interpreted using the model. Subsequently, model predictions based on this data were used to inform additional time-course experiments and analyse the results.

## 2 Methods

### 2.1 Transporter model

It was assumed that the kinetics of amino acid transport across the placental MVM could be described by a carrier-mediated process (Friedman, 2008, Stein and Lieb, 1986, Turner, 1983). An amino acid cannot traverse the cell membrane on its own, but needs to bind to a specific transport protein (Cleal and Lewis, 2008). Once the amino acid is bound, the transporter (carrier) can undergo a conformational change, exposing the substrate binding site to the other side of the plasma membrane to allow for transport across. Depending on the assumptions made, the carrier model can represent both amino acid transport mediated by obligatory exchangers, as well as non-obligatory (facilitative) transporters.

**Fig. 1:**
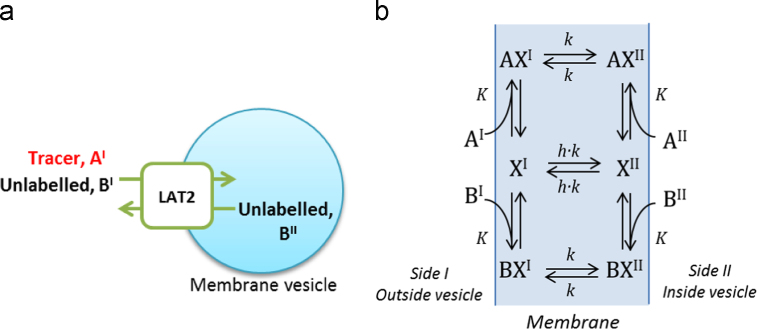
Overview of the experimental system and transporter model. (a) Transporter activity was evaluated by measuring the uptake of a radiolabelled tracer A into placental microvillous membrane vesicles. (b) Transporter model, schematic showing the different transporter states. Transporter X can bind to either tracer A or unlabelled substrate B to form a complex AX or BX, which can then translocate between the outside (I) and inside (II) of the vesicle plasma membrane. Transporter X can also translocate on its own, depending on the parameter h. The case h=0 corresponds to an obligatory exchanger, while for h≠0 the transporter will display facilitative diffusion.

An extensive treatment of carrier models can be found in the reference work by Stein (Stein and Lieb, 1986). Clarification of the underlying assumptions that apply to our model is presented in Appendix A. An overview of the current model is presented in Fig. 1. Radiolabelled substrate was used experimentally to measure uptake, while unlabelled substrate can either be present inherently, or added as part of the experimental design. Therefore, radiolabelled substrate A and unlabelled substrate B were distinguished explicitly in the model. The transporter, designated as unbound carrier X, can adopt two alternative states I and II, with a binding site exposed either on the outside I or inside II of the membrane. Amino acids A and B can bind reversibly to the transporter X to form a bound substrate-carrier complex, AX or BX, which itself can also alternate between the outside and inside of the plasma membrane (Fig. 1). It was assumed that each carrier could only bind a single amino acid molecule at any one time (Fotiadis et al., 2013).

A number of simplifying assumptions were made to reduce the number of parameters to the lowest possible to represent the main features of the proposed transport mechanism. The radiolabelled amino acid A, and unlabelled amino acid B were assumed to have identical transport characteristics. The translocation rate constants for the loaded transporter complex were assumed to be equal in forward and backward directions, both given by the rate constant k. This then also implied the same binding affinity on the inside and outside of the membrane, i.e. equal dissociation constants *K*, based on thermodynamic arguments (Appendix A). The bound and unbound carriers do not necessarily transfer at the same rate. Therefore, in addition, a parameter *h* was introduced to represent the relative mobility of the unbound carrier X with respect to the bound carrier complex AX or BX.

This parameter h is critical in distinguishing obligatory exchange from non-obligatory transport. As can be observed from Fig. 1, for h=0, the unbound carrier does not move on its own and only the bound carrier complex can translocate. Therefore transport is a perfectly obligatory exchange process in which A needs to be exchanged for B on a 1:1 basis for any net transport to occur. In contrast, if h is not zero then the unbound carrier can move on its own. Therefore the process is non-obligatory, as no substrate is required on the other (trans) side of the membrane in order for the carrier to return and continue the transport cycle. Thus for h>0 this can give rise to facilitated diffusion. Based on the assumptions described earlier and in Appendix A, the following equation can be derived for the uptake of radiolabelled substrate A into the vesicle:

(1) $$\frac{d{\left[A\right]}^{\mathrm{II}}}{dt}=\frac{2V\left({\left[A\right]}^{I}({\left[B\right]}^{\mathrm{II}}+Kh)-{\left[A\right]}^{\mathrm{II}}({\left[B\right]}^{I}+Kh)\right)}{2{\left[Tot\right]}^{I}{\left[Tot\right]}^{\mathrm{II}}+K\left(h+1\right)\left({\left[Tot\right]}^{I}+{\left[Tot\right]}^{\mathrm{II}}\right)+2hK^{2}}$$

where

$${\left[Tot\right]}^{i}={\left[A\right]}^{i}+{\left[B\right]}^{i}\;,\;\mathrm{for}\;i=I,\text{II}$$

Here h is dimensionless, K has unit of concentration (µmol l^−1^) and the uptake rate V is in µmol l^−1^ min^−1^. Note that V is in concentration per unit time as it incorporates the vesicle volume (i.e. flux in mol min^−1^ divided by vesicle volume). Thus from Eq. (1), obligatory exchange and non-obligatory or facilitative transport are essentially based on the same model, depending on the value of *h*.

### 2.2 Michaelis–Menten interpretation

In most experimental vesicle studies only the initial uptake rate is measured, with no amino acids added inside the vesicle (zero-trans conditions). Assuming Michaelis–Menten kinetics, uptake is then usually quantified in terms of the parameters $V_{\max}$ and $K_{m}$, i.e. the maximum uptake rate and half maximum rate concentration respectively. If we now consider Eq. (1) with no tracer inside at the start of the experiment ${\left[A\right]}_{0}^{\mathrm{II}}=0$, then the initial rate of tracer uptake can be written in the following form:

(2) $${\frac{d{\left[A\right]}^{\mathrm{II}}}{dt}}_{\left(t=0\right)}=\frac{V_{app}{\left[A\right]}_{0}^{I}}{{\left[Tot\right]}_{0}^{I}+K_{app}}$$

where

(3) $$V_{app}=\frac{2V\left({\left[B\right]}_{0}^{\mathrm{II}}+Kh\right)}{2{\left[B\right]}_{0}^{\mathrm{II}}+K\left(h+1\right)}$$

(4) $$K_{app}=\frac{K\left(h+1\right){\left[B\right]}_{0}^{\mathrm{II}}+2hK^{2}}{2{\left[B\right]}_{0}^{\mathrm{II}}+K\left(h+1\right)}$$

Thus, for initial uptake the model reduces indeed to a Michaelis–Menten relationship, where $V_{app}$ and $K_{app}$ are the apparent Michaelis–Menten parameters. However, critically it can be observed from Eqs. (3), (4) that these apparent Michaelis–Menten parameters depend directly on the value of the parameter h, as well as the concentration of any unlabelled substrate ${\left[B\right]}_{0}^{\mathrm{II}}$ that may be present inside the vesicle. Therefore, Eq. (2) can be applied to interpret apparent uptake parameters for either obligatory or non-obligatory exchange.

### 2.3 Experimental kinetics of ^14^C-serine uptake into MVM vesicles

Placentas were obtained following written informed consent with approval of the Central Manchester Research Ethics Committee (REC 12/NW/0574) from uncomplicated singleton pregnancies at term (38–40 weeks gestation) delivered by Caesarean section. MVM vesicles were isolated from each placenta using Mg^2+^ precipitation and differential centrifugation (Glazier and Sibley, 2006, Glazier et al., 1988). The final pellet was resuspended in intravesicular buffer (IVB; 290 mmol l^−1^ sucrose, 5 mmol l^−1^ HEPES, 5 mmol l^−1^ Tris, pH 7.4). MVM fragments were vesiculated by passing 15 times through a 25-gauge needle and stored at −80 °C prior to use. ^14^C-serine uptake (zero-trans) experiments were performed under sodium free conditions as previously described (Lewis et al., 2007). MVM vesicles (diluted to a protein concentration of 10 mg/ml with IVB) were equilibrated to room temperature (21–25 °C) prior to uptake. Uptake of ^14^C-serine into MVM vesicles was initiated by the addition of 20 µl MVM vesicle suspension to 20 µl extravesicular buffer (EVB; 5 mmol l^−1^ HEPES, 5 mmol l^−1^ Tris, 145 mmol l^−1^ KCl, pH 7.4) containing ^14^C-serine. An extravesicular tracer concentration of 7.5 µmol l^−1^ ^14^C-serine after dilution was used throughout the study. Uptake of 7.5 µmol l^−1^ ^14^C-serine was confirmed to be linear for up to 15 s for additional unlabelled serine concentrations of (0, 10, 50, 100, 1000 and 2000 μmol l^−1^), consistent with previous reports (Lewis et al., 2007), while uptake for higher concentrations was not significantly different from zero. To determine the initial uptake rate, tracer uptake into the vesicles was measured at *t*=15 s. The experiment was stopped by the addition of 2 ml ice-cold Krebs buffer (130 l^−1^ NaCl, 10 l^−1^ Na_2_HPO_4_, 4.2 l^−1^ KCl, 1.2 l^−1^ MgSO_4_, 0.75 l^−1^ CaCl_2_, pH 7.4) and filtered through a 0.45 µm nitrocellulose filter under vacuum. Filters were washed with 10 ml Krebs buffer and the filter-associated radioactivity was determined by liquid scintillation counting. To determine the kinetics of ^14^C-serine uptake into MVM vesicles, uptake of (extravesicular) 7.5 µmol l^−1^ ^14^C-serine was determined in the presence of increasing extravesicular concentrations of unlabelled serine (0.2 µmol l^−1^–20 l^−1^). For each placenta (*n*=4), individual measurements were performed at 23 different serine concentrations within this range.

In addition, time series experiments were conducted by measuring tracer uptake for placentas (*n*=3) at specific time points (0, 5, 10, 15, 20, 60, 120, 300, 600 s), for extravesicular concentrations of unlabelled serine of 0, 250 and 1000 μmol l^−1^.

### 2.4 Numerical implementation

Model equations were implemented in Matlab (R2013a). To predict the concentration within the vesicle over time, time series were generated by integrating Eq. (1) using the ode45 function (Runge–Kutta (4, 5) method). Apparent Michaelis–Menten parameters were determined by fitting Eq. (2) to the averaged experimental uptake rate measurements, using a least square criterion and the fminsearch function (Nelder-Mead simplex method). An intravesicular volume of 1.6 μl mg protein^−1^ was used to convert tracer uptake from units of mol per mg protein to concentration in mol per unit volume. This volume conversion factor was based on the previously measured equilibrium Na^+^ uptake (at 60 min) of 1.60±0.20 nmol mg protein^−1^ (mean±SEM, *n*=6), at a Na^+^ substrate concentration of 1 nmol µl^−1^, obtained using MVM vesicles prepared by the same method (Glazier et al., 1988).

The same methodology was used to fit the time course experiments. A single set of parameters h and V was fitted to represent all experimental conditions, as specified further in the results. To account for the difference in absolute values between experimental conditions, for each data point the difference between model prediction and experiment was normalised first by the experimental value, squared and then summed over all points to yield the overall error criterion to be minimised. A sensitivity analysis was carried out to determine the impact of individual parameters on the fit quality by fixing these parameters at a range of different levels and then repeating the full parameter estimation procedure for each.

## 3 Results

### 3.1 Model behaviour—Initial uptake rate

**Fig. 2:**
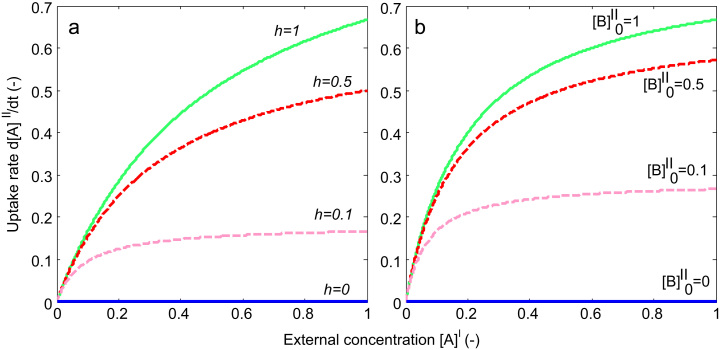
Initial uptake rate of tracer A as a function of its external concentration ${\left[A\right]}^{I}$. (a) Non-obligatory transport for different values of h, with zero internal substrate. (b) Obligatory exchanger (h=0) for different concentrations of unlabelled substrate ${\left[B\right]}^{\mathrm{II}}$ inside the vesicle (arbitrary units). Both hypotheses display qualitatively similar Michaelis–Menten type behaviour.

Generic model behaviour (Eq. (2)) is illustrated in Fig. 2 for parameters V=1 and K=0.5. It can be observed that both non-obligatory and obligatory exchange can give rise to Michaelis–Menten type behaviour (i.e. linear uptake for low and constant uptake for high external concentrations at saturation), depending on conditions. For the non-obligatory model in Fig. 2a, the initial uptake rate of tracer A was calculated under zero trans conditions, i.e. internal concentrations were set to zero ${\left[A\right]}_{0}^{\mathrm{II}}=0$ and ${\left[B\right]}_{0}^{\mathrm{II}}=0$. In addition, for clarity the external unlabelled concentration was also set to zero ${\left[B\right]}_{0}^{I}=0$. Under these conditions, uptake as a function of external tracer concentration ${\left[A\right]}_{0}^{I}$ displayed Michaelis–Menten behaviour depending on the value of h. The case h=0 corresponds to obligatory exchange and therefore did not show any uptake in the absence of internal concentrations, as expected. Similarly, in Fig. 2b, results are reported for the case of obligatory exchange (h=0), but now assuming the presence of different concentrations of unlabelled concentrations ${\left[B\right]}_{0}^{\mathrm{II}}$ inside the vesicle, as an internal concentration of amino acids is a perquisite for obligatory exchange (${\left[A\right]}_{0}^{\mathrm{II}}=0$ and ${\left[B\right]}_{0}^{I}=0$, as before). It can be seen that the initial uptake rate as a function of external concentration displayed a strong dependence on internal concentration, with a significant uptake rate already reached for relatively low internal concentrations, e.g. ${\left[B\right]}_{0}^{\mathrm{II}}=0.1$. Again, for the case ${\left[B\right]}_{0}^{\mathrm{II}}=0$ no uptake took place, as would be expected for an obligatory exchanger.

### 3.2 Model behaviour—Time series

**Fig. 3:**
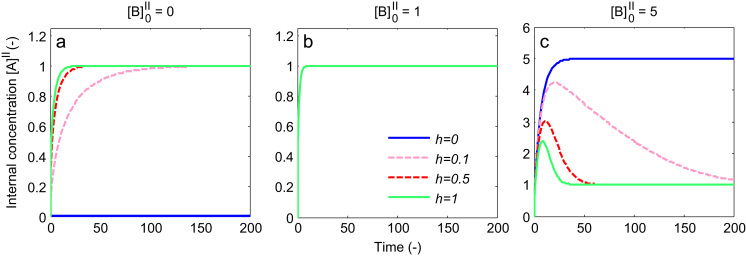
Theoretical model behaviour for obligatory exchange (h=0)and facilitated transport (h>0). Time series for different values of h (relative mobility of the unloaded transporter) and initial concentrations ${\left[B\right]}_{0}^{\mathrm{II}}$ inside the vesicle (arbitrary units). The external concentration of tracer ${\left[A\right]}^{I}=1$ in all cases. (a) Zero initial substrate inside; note that no uptake takes place for obligatory exchange. (b) Equal internal and external concentrations; note all lines overlap. (c) Higher internal concentration; note the overshoot dependent on the value of h, with h=0 corresponding to obligatory exchange and h>0 to facilitated diffusion.

In order to differentiate non-obligatory vs obligatory exchange behaviour, a potential time series experiment was simulated (Fig. 3), for a combination of different values of h and internal concentration levels ${\left[B\right]}_{0}^{\mathrm{II}}$. It was assumed the extravesicular buffer volume is large enough so that external concentrations could be assumed constant. Therefore, a fixed value of external tracer ${\left[A\right]}^{I}=1$ and unlabelled concentration ${\left[B\right]}^{I}=0$ were used in Eq. (1), with the same parameters V=1 and K=0.5. The initial tracer inside the vesicle ${\left[A\right]}_{0}^{\mathrm{II}}=0$. Fig. 3a shows that in the absence of initial internal concentrations $\left({\left[B\right]}_{0}^{\mathrm{II}}=0\right)$, uptake followed a facilitated diffusion process, in which the predicted tracer concentration within the vesicle rises up to an equilibrium level equal to the external concentration. The rate at which this equilibrium is reached increases with increasing h, while no uptake is observed for the obligatory exchanger (h=0), as before.

However, Fig. 3b shows that no difference between the obligatory and non-obligatory models could be observed for the case (${\left[B\right]}_{0}^{\mathrm{II}}=1)$ where the initial internal concentration is equal to the external tracer concentration. Furthermore, predictions were not sensitive to the value of h, i.e. note that all lines in the figure overlap.

In contrast, Fig. 3c shows clear differences between the obligatory and non-obligatory model for the case of high internal concentrations (${\left[B\right]}_{0}^{\mathrm{II}}=5$). For the obligatory exchanger (h=0) the tracer concentration increases monotonically until an equilibrium is reached, which is higher than the external concentration. This is because at the end of the experiment all internal unlabelled substrate present initially $\left({\left[B\right]}_{0}^{\mathrm{II}}\right)$ has been replaced by tracer ${\left[A\right]}^{\mathrm{II}}$ (i.e. as a result of continued 1:1 exchange with only tracer present externally and unlabelled substrate assumed to dilute out, ${\left[B\right]}^{I}=0$ as stated previously).

More complex transient behaviour is observed for the non-obligatory model (h≠0) in Fig. 3c. The large outwardly directed gradient of unlabelled substrate initially drives tracer uptake via exchange, resulting in a substantial overshoot, which then goes back to the diffusive equilibrium as the outwardly directed gradient dissipates. The rate at which the tracer concentration returned to the diffusive equilibrium depended directly on the value of h, i.e. the mobility of the unloaded carrier. Thus for low values of h the transporter primarily behaved as a 1:1 exchanger initially, before returning to equilibrium more slowly via facilitated diffusion.

### 3.3 Model interpretation of serine uptake experiment

**Fig. 4:**
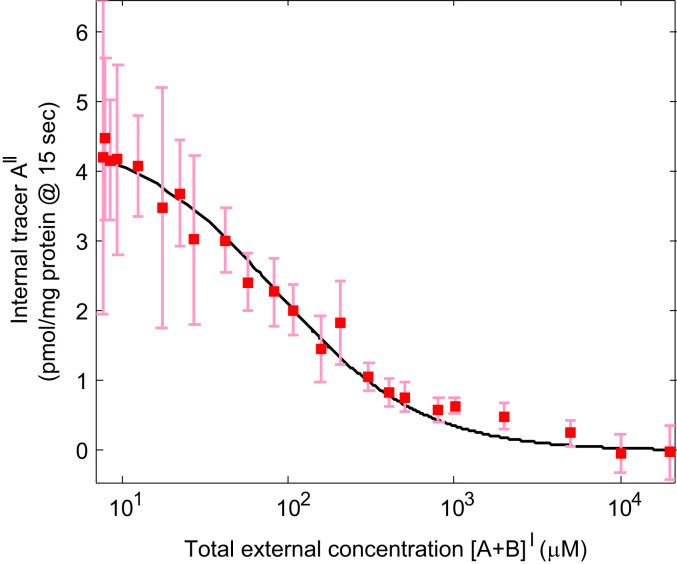
Results vesicle experiments: Initial tracer uptake as a function of the total external concentration (labelled+unlabelled substrate). 7.5 µmol l^−1^ tracer (^14^C-serine) was used throughout, while the concentration of unlabelled serine was increased. Data presented as mean and standard deviation (*n*=4). Experimental results were fitted using Eq. (2) (solid black line).

Next, to relate the model to the experimental data, the tracer uptake measurements were fitted using Eq. (2) as outlined in Section 2 (Fig. 4), resulting in $K_{app}=\;87\;µ\text{mol}\;l^{-1}$ and $V_{app}=\;131\;µ\text{mol}\;l^{-1}\;\min^{-1}$. Subsequently, Eqs. (3), (4) were used to determine the actual values of the model parameters K and V that would correspond to these apparent Michaelis–Menten parameters for different values of h and ${\left[B\right]}_{0}^{\mathrm{II}}$, (Table 1). In general K and V were higher than the apparent parameters $K_{app}$ and $V_{app}$, but values decreased for increasing internal serine concentration present. Increasing h also reduced K and V, and in addition decreased the sensitivity to the internal serine concentration. For h=1 the model parameters became independent of any internal serine concentration present, and moreover K and V corresponded directly to the apparent Michaelis–Menten parameters $K_{app}$ and $V_{app}$.

**Table 1:** Model parameters (Eq. (1)) corresponding to the measured apparent Michaelis–Menten parameters, for different unloaded carrier mobility h and internal concentration ${\left[B\right]}_{0}^{\mathrm{II}}$.

	${\left[B\right]}_{0}^{\mathrm{II}}=0\;µ\text{mol}\;l^{-1}$	${\left[B\right]}_{0}^{\mathrm{II}}=250\;µ\text{mol}\;l^{-1}$	${\left[B\right]}_{0}^{\mathrm{II}}=500\;µ\text{mol}\;l^{-1}$	${\left[B\right]}_{0}^{\mathrm{II}}=1000\;µ\text{mol}\;l^{-1}$
h=0	No uptake^a^	K=266 µmol l^−1^	K=210 µmol l^−1^	K=190 µmol l^−1^
		V=201 µmol l^−1^ min^−1^	V=159 µmol l^−1^ min^−1^	V=144 µmol l^−1^ min^−1^
h=0.1	K=478 µmol l^−1^	K=198 µmol l^−1^	K=177 µmol l^−1^	K=167 µmol l^−1^
	V=722 µmol l^−1^ min^−1^	V=175 µmol l^−1^ min^−1^	V=152 µmol l^−1^ min^−1^	V=141 µmol l^−1^ min^−1^
h=0.5	K=130 µmol l^−1^	K=119 µmol l^−1^	K=118 µmol l^−1^	K=117 µmol l^−1^
	V=197 µmol l^−1^ min^−1^	V=144 µmol l^−1^ min^−1^	V=138 µmol l^−1^ min^−1^	V=135 µmol l^−1^ min^−1^
h=1	K=87 µmol l^−1^	K=87 µmol l^−1^	K=87 µmol l^−1^	K=87 µmol l^−1^
	V=131 µmol l^−1^ min^−1^	V=131 µmol l^−1^ min^−1^	V=131 µmol l^−1^ min^−1^	V=131 µmol l^−1^ min^−1^

a The case h=0 corresponds to an obligatory exchanger and therefore cannot represent uptake in the absence of ${\left[B\right]}_{0}^{\mathrm{II}}$.

### 3.4 Model predictions based on experimental parameters

**Fig. 5:**
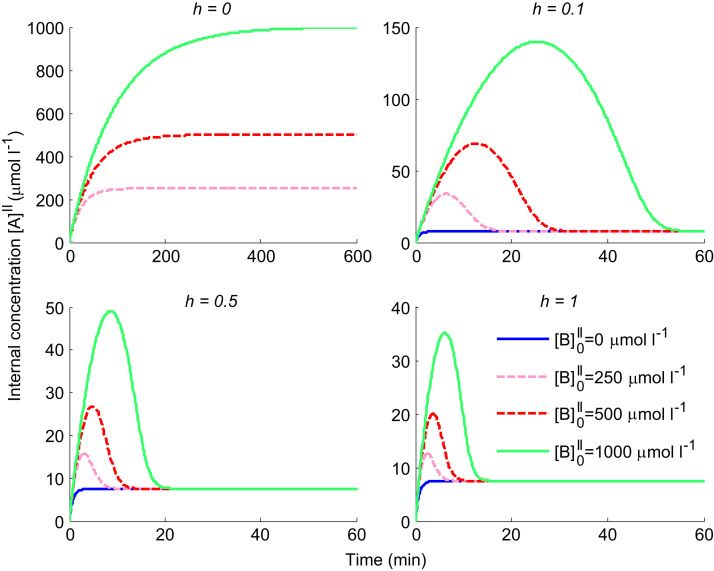
Model predictions based on the experimental initial uptake data. Time series were generated for the various scenarios, including different values of h, internal substrate concentrations ${\left[B\right]}_{0}^{\mathrm{II}}$ and model parameters based on the experimental measurements (Table 1). Note the difference in timescale for h=0 (top left).

Subsequently, the model was used to predict the uptake behaviour that could be expected if the experiment were continued beyond the initial phase. Fig. 5 shows the time series results generated using the model parameters derived in Table 1 for the corresponding initial conditions and values of h. For the obligatory exchanger (h=0) tracer concentrations continued to rise to very high values, until equilibrium was reached at the level of the initial unlabelled concentration present inside ${\left[B\right]}_{0}^{\mathrm{II}}$ (i.e. all unlabelled substrate replaced by labelled on a 1:1 basis). For non-zero h in all cases, internal tracer concentrations eventually reached a diffusive equilibrium equal to the external tracer concentration of 7.5 µmol l^−1^.

For zero initial internal concentration $\left({\left[B\right]}_{0}^{\mathrm{II}}=0\right)$, the tracer concentrations rose monotonically, while for non-zero initial concentrations ${\left[B\right]}_{0}^{\mathrm{II}}$, first an overshoot was produced due to trans-stimulation before decreasing again to the diffusive equilibrium level. In addition, the peak height and rate at which the concentration returned to equilibrium were determined by the value of h, i.e. the relative mobility of the unloaded transporter.

### 3.5 Time course experiments

**Fig. 6:**
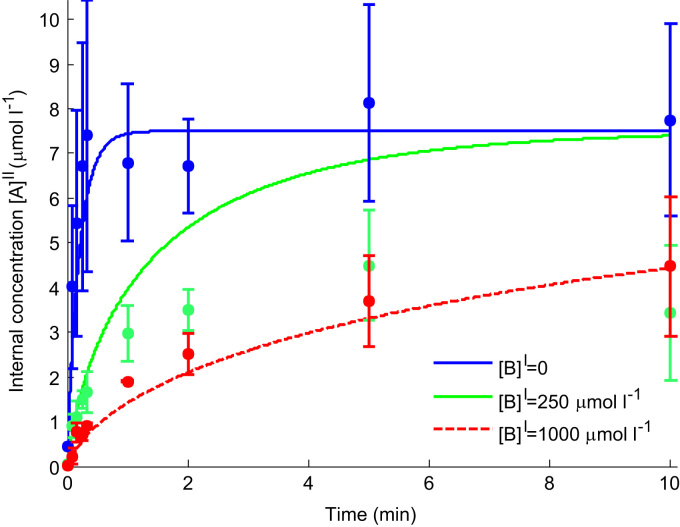
Time course results for the uptake of 7.5 µmol l^−1^ tracer (^14^C-serine) in the vesicle for various concentrations of additional external unlabelled serine ${\left[B\right]}^{I}$. Experimental data is presented as mean and standard deviation (*n*=3). Lines represent the model fit for a facilitated diffusion transport process (h>0) in the absence of initial internal concentrations in the vesicle $\left({\left[B\right]}_{0}^{\mathrm{II}}=0\right)$. Note that facilitated diffusion implies that at steady state the tracer concentrations inside and outside the vesicle are equal.

The results of the time course experiments are presented in Fig. 6. Uptake of tracer alone displayed a rapid rise in internal concentrations up to constant steady state level. Given the experimental variability, the existence of a small overshoot could neither be confirmed nor excluded. The internal tracer concentration at equilibrium (based on the volume conversion factor of 1.6 μl mg protein^−1^) was approximately equal to the external tracer concentration of 7.5 µmol l^−1^.

To further probe the transport behaviour, additional experiments were performed in which either 250 or 1000 µmol l^−1^ of unlabelled substrate was added to the extravesicular buffer. As can be observed from Fig. 6, addition of external unlabelled substrate resulted in a marked inhibition of the initial rate of tracer uptake, dependent on the amount of substrate added. After the initial phase, intravesicular tracer levels in the presence of either 250 or 1000 µmol l^−1^ external substrate continued to rise at a decreasing rate, but did not reach the level observed for tracer alone within the time frame of the study.

### 3.6 Model analysis of time course experiments

First it was evaluated how well the model could represent the experimental data in absence of internal endogenous substrate $\left({\left[B\right]}_{0}^{\mathrm{II}}=0\right)$. A single set of parameters h and V were fitted, while K was derived using Eq. (4) from the value of $K_{app}=87\;µ\text{mol}\;l^{-1}$ determined previously in Section 3.3.

The model results in Fig. 6 show a good overall representation of the initial tracer uptake for the various external concentrations of unlabelled substrate (${\left[B\right]}^{I}=$0, 250 and 1000 µmol l^−1^). Furthermore, the time course for 0 and 1000 µmol l^−1^ was captured adequately. The main discrepancy that can be observed is the considerable overprediction of uptake for 250 µmol l^−1^ unlabelled substrate from 60 s onwards.

**Fig. 7:**
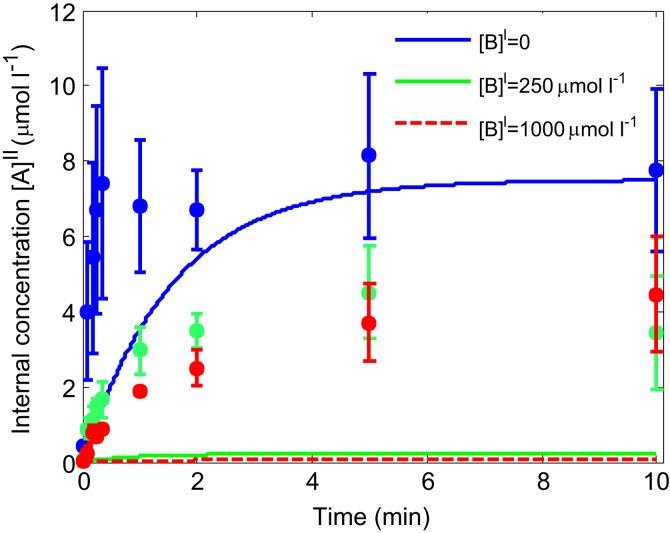
Time course for an obligatory exchanger (h=0). Lines represent the model fit in the presence of initial internal substrate ${\left[B\right]}_{0}^{\mathrm{II}}=\;7.5\;µ\text{mol}\;l^{-1}$. Experimental data (same as in Fig. 6) is presented as mean and standard deviation (*n*=3). Note that while the experimental equilibrium tracer level for zero external unlabelled serine $\left({\left[B\right]}^{I}=0\right)$ could be matched, the model predicted a disproportional reduction in uptake for additional external unlabelled serine of 250 and 1000 µmol l^−1^.

In addition, the possibility was investigated of obligatory exchange (h=0) in the presence of a small internal level of substrate ${\left[B\right]}_{0}^{II}=7.5\;\mu \text{mol}\;l^{-1}$, equal to the external tracer concentration. Only the parameter V was fitted, while K was kept the same value as determined before in Fig. 6. The results in Fig. 7 show that while obligatory exchange could match the equilibrium level for tracer alone, it could not simultaneously represent the effect of adding unlabelled external substrate, as this would result in disproportionally low levels of intravesicular tracer.

**Fig. 8:**
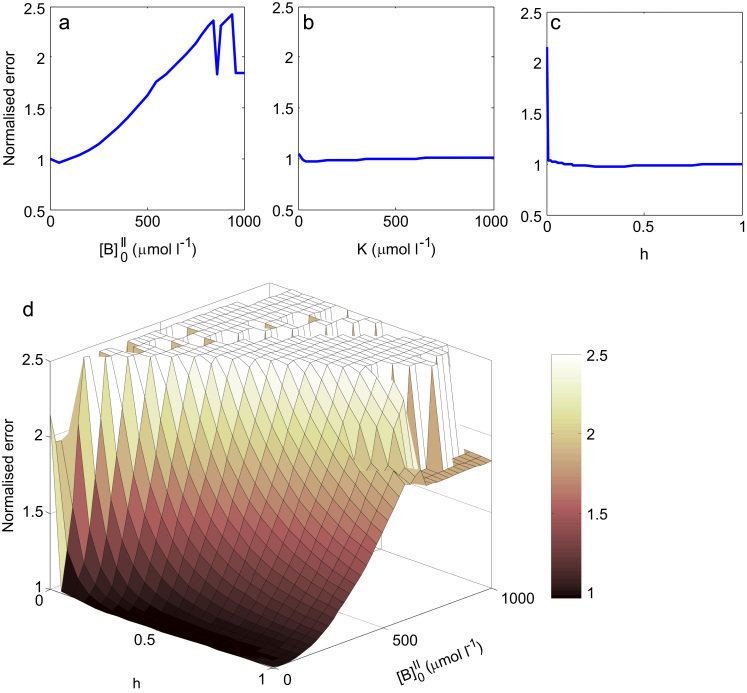
Sensitivity analysis. The model fit was repeated for a range of fixed parameters and the overall error expressed relative to the fit in Fig. 6. (a) Effect of initial internal concentration ${\left[B\right]}_{0}^{\mathrm{II}}$, (b) dissociation constant K, and (c) relative mobility of the unloaded transporter h. Note the sharp peak for h=0, while the error is approximately constant for any h>0. (d) Combined effect of h and ${\left[B\right]}_{0}^{\mathrm{II}}$ on the fit quality (values >2.5 have been omitted). Note the error is more sensitive to ${\left[B\right]}_{0}^{\mathrm{II}}$ for low values of h.

To provide a more complete overview, the results of the sensitivity analysis are presented in Fig. 8. The model fit as in Fig. 6 was repeated, but now for various levels of initial internal substrate ${\left[B\right]}_{0}^{\mathrm{II}}$ (parameters h and V were fitted, with K determined from Eq. (4) as before). Overall the relative error increased with increasing internal substrate concentration (Fig. 8a). A very small dip in the error criterion could be observed for ${\left[B\right]}_{0}^{\mathrm{II}}=50\;µ\text{mol}\;l^{-1}$, however investigation of the predicted time course already displayed an overshoot not present in the experiment (results not shown). Next the sensitivity of the parameter estimation to the dissociation constant K was investigated (parameters h and V were fitted with ${\left[B\right]}_{0}^{\mathrm{II}}=\;0\;µ\text{mol}\;l^{-1}$). The results in Fig. 8b demonstrated that the overall error was not significantly affected by K. Finally, the effect of the unbound carrier mobility was explored in Fig. 8c by repeating the fit for various fixed values of h (only the parameter V was fitted with ${\left[B\right]}_{0}^{\mathrm{II}}=\;0\;µ\text{mol}\;l^{-1}$, while K was kept the same value as determined before in Fig. 6). This demonstrated that h did not have a significant impact on the overall error, with the notable exception of a sharp peak at h=0. Looking closer at the estimated parameters revealed that low values of h resulted in high values of V and vice versa, but the shape of the curves was qualitatively similar for any h>0 (results not shown). The combined effect of h and ${\left[B\right]}_{0}^{\mathrm{II}}$ on the fit quality is displayed in Fig. 8d, and shows that the overall error is more sensitive to ${\left[B\right]}_{0}^{\mathrm{II}}$ for low values of h. For high concentrations the error is highly variable as the fitted time courses cannot represent the experimental data (results not shown).

## 4 Discussion

Placental membrane vesicle experiments are used routinely for the study of exchanger transporters (Ganapathy et al., 1986, Jansson et al., 1998, Lewis et al., 2007, Tsitsiou et al., 2009). However, these experiments consistently display substrate uptake under zero-trans conditions, which is incompatible with the concept of an obligatory exchanger mechanism and remains currently unexplained. Therefore, mathematical modelling was applied to clarify how these experimental observations could relate to the behaviour of the transporter under investigation. Importantly, for clarity and to facilitate biological interpretation the number of parameters in the model was kept to the minimum required to represent the key transporter mechanisms at play.

Both non-obligatory and obligatory exchanger models could explain the observed initial rate of serine tracer uptake under sodium free conditions, depending on the initial substrate concentration inside the vesicle. Previous evidence for an obligatory exchanger, as opposed to facilitated diffusion, is provided by the 1:1 ratio between amino acid influx and efflux, combined with the observation that no significant amino acid depletion was detected in oocytes maintained in amino acid free buffer (Meier et al., 2002). However, for an obligatory exchanger to function this would necessarily imply the presence of initial substrate within the vesicle. In this respect, the dependency of apparent Michaelis–Menten parameters on internal substrate concentration derived in the current study (Eqs. (3), (4)), could potentially help explain variation in reported $K_{m}$ values for Na^+^ independent serine uptake in the literature (116–675 µmol l^−1^) (Lewis et al., 2007, Segawa et al., 1999). However, the internal concentrations cannot affect the apparent $K_{m}$ if the unloaded and loaded transporters have equal mobility (h=1) in the carrier model (Eq. (4), Table 1; i.e. since binding of internal substrate would not affect the return rate of the carrier in the transport cycle).

Since a standard uptake experiment did not allow non-obligatory and obligatory exchange to be distinguished based on initial rate alone (Fig. 2a and b), the possibility of continuing the experiment as a time series was explored first theoretically and then tested experimentally. Based on the model predictions in Fig. 5, the lack of a pronounced overshoot observed in the experimental time course (Fig. 6) indicated the absence, or only low levels, of internal substrate within the vesicles. In addition, equal internal and external tracer concentrations at steady state were observed in the experiment (Fig. 6), which provided a strong indication for non-obligatory transport leading to diffusive equilibrium. Furthermore, tracer uptake could be completely inhibited (Fig. 4), demonstrating that uptake was transporter mediated as opposed to simple diffusion, within experimental accuracy. However, similar concentrations on both sides of the membrane are not conclusive proof of facilitated diffusion, as an obligatory exchanger would give the same result in the case where internal and external concentrations were equal (Fig. 3b). In addition, the vesicle volume conversion factor used here to determine internal concentration levels may not be known precisely in many cases. Therefore, the time course experiment was repeated in the presence of additional external unlabelled serine. This showed that the experimental data could be captured reasonably well by the model based on facilitated diffusion (Fig. 6), while obligatory exchange would predict a disproportionate reduction in steady state concentrations (Fig. 7). This is because at equilibrium, obligatory exchange would result in equal ratios of tracer to unlabelled substrate inside and outside the vesicle, and 7.5 µmol l^−1^ tracer represents only a very small fraction compared to either 250 or 1000 µmol l^−1^ unlabelled substrate.

Overall, the results of this study supported the existence of a facilitative transport component in placental MVM vesicles. However, while h was clearly non-zero in the model, the results of the sensitivity analysis demonstrated that it was not possible to ascertain the precise value of h with confidence. This is because for a facilitative transporter, in absence of significant internal concentrations, the tracer uptake depends on the combined velocity of the unloaded and loaded transporter in the transport cycle, thus h and V could not be determined independently. Based on previous exchange and efflux experiments in oocytes one would expect h to be low (Meier et al., 2002). A relatively slow unloaded transporter (small h) would increase the potential for trans-stimulation of the initial uptake rate by internal substrate (Harrison and Christensen, 1975) and display a larger overshoot (Fig. 5). Thus further opportunities to determine h could be provided by developing experimental protocols in which additional high concentrations of substrate are added within the vesicle. In addition, the presence of an overshoot in such an experiment would directly demonstrate a non-obligatory transport mechanism (Fig. 3, Fig. 5). This is because a high outwardly directed substrate gradient would promote an overshoot response for non-obligatory transport (Fig. 5b–d), while an obligatory exchanger would be expected to equilibrate at different levels directly dependent on the concentration added inside the vesicle (Fig. 5a). Time course data for the sodium independent uptake of system L substrates in non-preloaded MVM vesicles displayed a small overshoot for tryptophan, as indicated by the drop in tracer level at 45 min (Ganapathy et al., 1986), while in contrast, no overshoot was observed for leucine (Jansson et al., 1998).

**Fig. B3:**
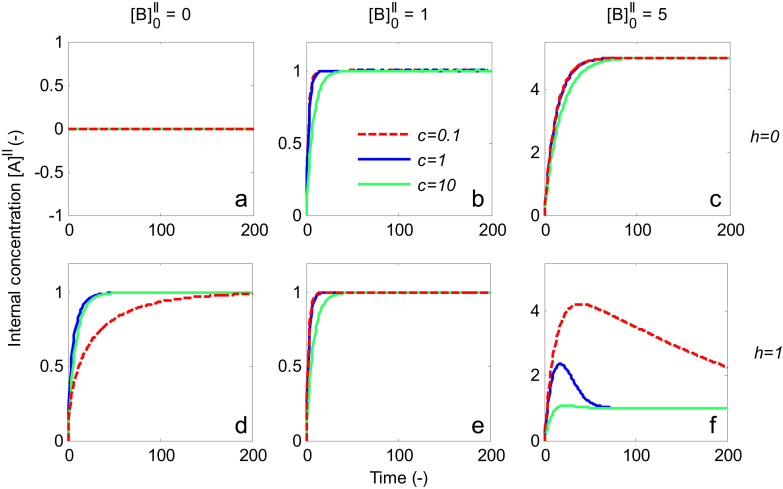
Effects of asymmetry in unbound transporter (X) translocation rate $\left(k_{2}\neq k_{-2}\right)$ and dissociation constants $\left(K_{I}\neq K_{\mathrm{II}}\right)$ according to Eqs. (B4), (B5). (a-c) Results for obligatory exchange, h=0. (d–f) Results for the non-obligatory model, with h=1. Time series for different values of c and initial concentrations ${\left[B\right]}_{0}^{\mathrm{II}}$ inside the vesicle (arbitrary units). The external concentration of tracer ${\left[A\right]}^{I}=1$ in all cases. (a) and (d) Zero initial substrate inside. (b) and (e) Equal internal and external concentrations. (c) and (f) Higher internal concentration.

A number of simplifying assumptions were made in the application of the model. In particular the transport parameters in the model were assumed to be symmetric. However, in Xenopus oocyte studies asymmetric apparent affinities have been found (Meier et al., 2002). This could be explained by different translocation rates and dissociation constants according to Eqs. (A9) and (A17). Nonetheless, importantly, relaxing these model assumptions would not affect the equilibrium substrate concentrations for a passive transport process. Membrane potential was not included as LAT2 is a sodium independent transporter of neutral amino acids and not known to be electrogenic (Broer, 2008; Ganapathy et al., 1988; Kanai et al., 1998; Pineda et al., 1999). The effect of asymmetry on the model predictions was explored further in Appendix B. This confirmed that the presence of an overshoot would indicate a non-obligatory as opposed to an obligatory exchange mechanism, however the reverse is not true as such an overshoot depends both on model parameters and internal concentrations (Fig. B3). An average vesicle volume conversion factor was used to determine intravesicular tracer concentrations, while vesicles in suspension appear heterogeneous in size (Glazier et al., 1988) and thus would display a range of area to volume ratios, potentially smoothing out the response. In addition, the vesicle volume was assumed to be constant over time, while any changes in volume might for example contribute to explaining the overprediction observed for 250 µmol l^−1^ external unlabelled serine in Fig. 6. Based on current understanding, a single transporter (LAT2) was proposed to represent the sodium independent transport of serine, and all known Na^+^ independent serine transport systems are exchangers. However, if multiple transport mechanisms were to be present (e.g. a previously unidentified facilitative serine transporter) this would give rise to a mixed response which would be more difficult to interpret, e.g. an obligatory exchanger in combination with an unknown diffusive transport route in parallel could give rise to a qualitatively similar response as a non-obligatory transporter.

In conclusion, the interpretation of vesicle experiments such as those with placental MVM in reality may be complicated by non-zero internal substrate concentrations. In addition, actual transporter behaviour may deviate from idealised behaviour such as perfectly obligatory exchange. Modelling could allow quantification of these effects in order to reveal their impact. However, to achieve this, an iterative approach is needed in which the model is used to predict various potential transport scenarios, which can then be tested experimentally, leading to model refinement and new experiments. In this way, modelling could help to interpret and design uptake experiments and contribute to a more complete understanding of the behaviour of specific transport systems.

## Figures and Tables

**Fig. A1:**
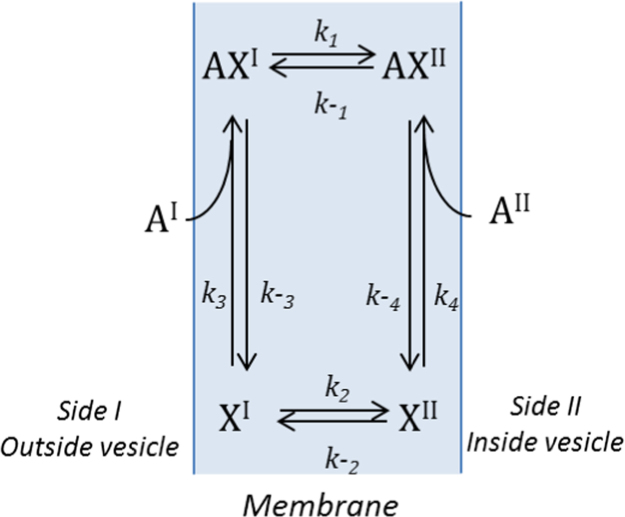
General carrier model for a single substrate. Schematic including the different transporter states and the various binding and translocation rate constants. Transporter X can bind to substrate A to form a complex AX. Depending on parameters, X can translocate between the outside (I) and inside (II) of the vesicle in either loaded or unloaded form.

**Fig. B1:**
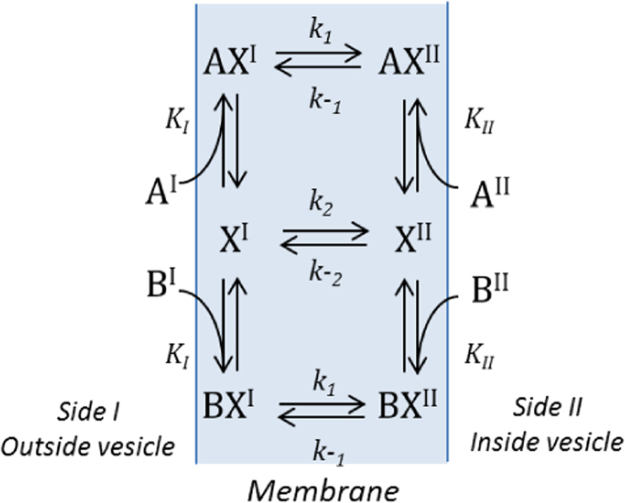
Extended carrier model for two substrates (A is radiolabelled, and B is unlabelled). Schematic including the different transporter states and the various dissociation and translocation rate constants. Transporter X can either bind to substrate A or B to form a complex AX or BX. X can translocate between the outside (I) and inside (II) of the vesicle in either loaded or unloaded form with different translocation rate constants in each direction.

**Fig. B2:**
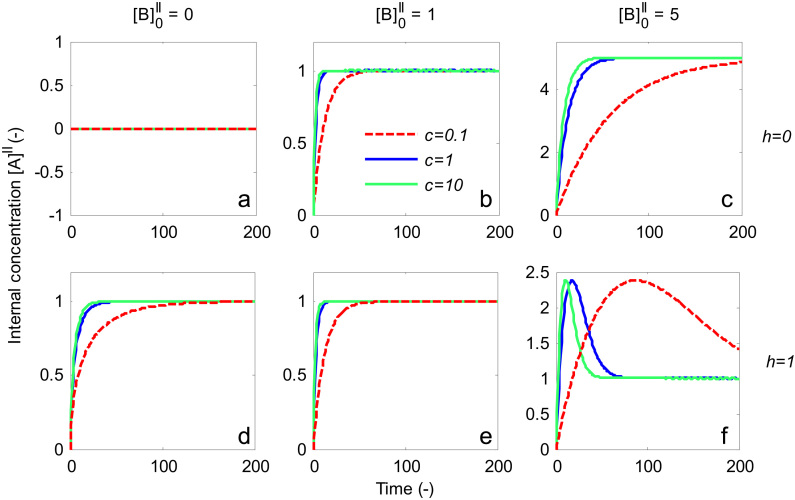
Effects of asymmetry in bound transporter (AX and BX) translocation rate $\left(k_{1}\neq k_{-1}\right)$ and dissociation constants $\left(K_{I}\neq K_{\mathrm{II}}\right)$ according to Eqs. (B2), (B3). (a–c) Results for obligatory exchange, h=0. (d–f) Results for the non-obligatory model, with h=1. Time series for different values of the asymmetry factor c and initial concentrations ${\left[B\right]}_{0}^{\mathrm{II}}$ inside the vesicle (arbitrary units). The external concentration of tracer ${\left[A\right]}^{I}=1$ in all cases. (a) and (d) Zero initial substrate inside. (b) and (e) Equal internal and external concentrations. (c) and (f) Higher internal concentration.

**Fig. B4:**
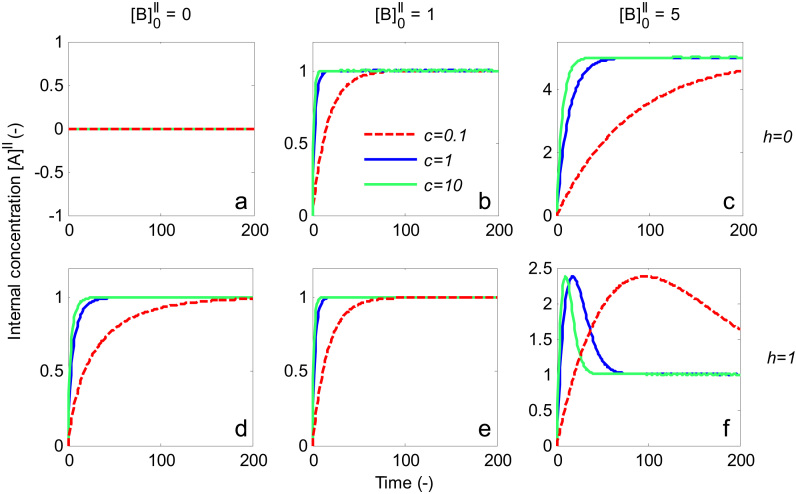
Effects of asymmetry in bound transporter (AX and BX) and unbound transporter (X) translocation rate constants ($k_{1}\neq k_{-1}$ and $k_{2}\neq k_{-2}$), with equal dissociation constants ($K_{I}=K_{\mathrm{II}}$), according to Eqs. (B6), (B7). Time series for different values of c and initial concentrations ${\left[B\right]}_{0}^{\mathrm{II}}$ inside the vesicle (arbitrary units). The external concentration of tracer ${\left[A\right]}^{I}=1$ in all cases. (a) and (d) Zero initial substrate inside. (b) and (e) Equal internal and external concentrations. (c) and (f) Higher internal concentration.

## Appendix A

A brief overview of the assumptions underlying the carrier model as applied in this study is provided (Friedman, 2008, Stein and Lieb, 1986). A schematic of the general carrier model is shown in Fig. A1.

The net flux $J_{AX}$ of the substrate-transporter complex AX from side I to side II, can be represented as the net effect of the forward and backward directed fluxes (Eq. (A1)), in which each flux is the product of the appropriate linear rate constant and concentration (Friedman, 2008). The same applies for the net flux $J_{X}$ of unbound transporter X from side I to II (Eq. (A2)).

(A1) $$J_{AX}=k_{1}{\left[AX\right]}^{I}-k_{-1}{\left[AX\right]}^{\mathrm{II}}$$

(A2) $$J_{X}=k_{2}{\left[X\right]}^{I}-k_{-2}{\left[X\right]}^{\mathrm{II}}$$

Rapid equilibrium is assumed, i.e. it is assumed that amino acid binding is fast compared to translocation across the membrane, which implies that bound and unbound concentrations are in equilibrium at each side of the membrane. Therefore, the rate constants for binding can be replaced by the following dissociation constants $K_{I}$ and $K_{\mathrm{II}}$:

(A3) $$K_{I}=\frac{{\left[A\right]}^{I}{\left[X\right]}^{I}}{{\left[AX\right]}^{I}}=\frac{k_{-3}}{k_{3}}$$

(A4) $$K_{\mathrm{II}}=\frac{{\left[A\right]}^{\mathrm{II}}{\left[X\right]}^{\mathrm{II}}}{{\left[AX\right]}^{\mathrm{II}}}=\frac{k_{-4}}{k_{4}}$$

Quasi-steady state transport is assumed, i.e. if the compartments on either side of the membrane are large enough, any changes in their concentrations will be relatively slow compared to the transporter dynamics. Thus quasi-steady transport implies that the sum of the fluxes of bound and unbound carrier must be zero, i.e. they must be equal and opposite for the transport cycle to continue:

(A5) $$J_{X}+J_{AX}=0$$

In addition, conservation of the total number of transporters $x_{T}$ is given by:

(A6) $${\left[X\right]}^{I}+{\left[X\right]}^{\mathrm{II}}+{\left[AX\right]}^{I}+{\left[AX\right]}^{\mathrm{II}}=x_{T}$$

Substrate A can only cross the membrane bound to the transporter, therefore $J_{A}\;=J_{AX}$. Then by rearranging Eqs. (A1), (A2), (A3), (A4), (A5), (A6), the general equation for the net flux of substrate A into the vesicle is given by:

(A7) $$J_{A}=\frac{\left(k_{-2}k_{1}K_{\mathrm{II}}{\left[A\right]}^{I}-k_{2}k_{-1}K_{I}{\left[A\right]}^{\mathrm{II}}\right)x_{T}}{\left(k_{1}+k_{-1}\right){\left[A\right]}^{I}{\left[A\right]}^{\mathrm{II}}+\left(k_{-2}+k_{1}\right)K_{\mathrm{II}}{\left[A\right]}^{I}+\left(k_{2}+k_{-1}\right)K_{I}{\left[A\right]}^{\mathrm{II}}+(k_{2}+k_{-2})K_{\mathrm{II}}\;K_{I}}$$

*Michaelis–Menten interpretation:* In experimental vesicle studies, uptake is usually reported in terms of apparent Michaelis–Menten parameters. Considering only the initial flux $J_{A_{0}}$, then all initial concentrations are known and for the case of zero-trans uptake, Eq. (A7) reduces to:

(A8) $$J_{A_{0}}=\frac{k_{-2}k_{1}{\left[A\right]}_{0}^{I}x_{T}}{\left(k_{-2}+k_{1}\right){\left[A\right]}_{0}^{I}+(k_{2}+k_{-2})\;K_{I}}$$

Therefore, the apparent Michaelis–Menten parameters can be expressed as follows (Devés and Krupka, 1979a, Devés and Krupka, 1979b):

(A9) $$K_{app}=\frac{\left(k_{2}+k_{-2}\right)}{\left(k_{-2}+k_{1}\right)}\;K_{I}$$

(A10) $$V_{app}=\frac{k_{-2}k_{1}x_{T}}{\left(k_{-2}+k_{1}\right)}$$

Thus $K_{app}$ is a function of the external substrate dissociation constant $K_{I}$, as well as the transporter translocation rates, while $V_{app}$ is a function of the translocation rates of the bound and unbound carrier.

*Equal translocation rates*: In order to reduce the number of parameters, it was assumed that the forward $\left(k_{i}\right)$ and backward $\left(k_{-i}\right)$ translocation rate constants as defined in Fig. A1 were equal (Eqs. (A11), (A12)). However, note that different rates could apply for the carrier substrate complex AX and the unbound transporter X:

(A11) $$k_{1}=k_{-1}=k$$

(A12) $$k_{2}=k_{-2}=hk$$

Here k represents the translocation rate constant for the bound carrier-substrate complex AX in either direction. Thus, h denotes the translocation rate of the unbound carrier X as a fraction of that of the carrier-substrate complex (i.e. the relative unbound-to-bound translocation rate). Therefore, the fluxes of the bound and unbound carrier become:

(A13) $$J_{AX}=k\left({\left[AX\right]}^{I}-{\left[AX\right]}^{\mathrm{II}}\right)$$

(A14) $$J_{X}=hk\left({\left[X\right]}^{I}-{\left[X\right]}^{\mathrm{II}}\right)$$

*Microscopic reversibility:* The transport mechanisms considered here are passive in nature, driven by concentration gradients only, without any energy input in the form of e.g. ATP. Therefore, the principle of microscopic reversibility implies that, for a cyclic transport process, the product of the clockwise rate constants is equal to the product of the anti-clockwise rate constants, Eq. (A16) (Blumenthal and Kedem, 1969, Parent et al., 1992) (i.e. based on the equilibrium of each step in the transport cycle at steady state and the known internal and external concentrations at equilibrium):

(A16) $$k_{1}\;k_{-4}\;k_{-2}\;k_{3}=k_{-1}\;k_{-3}\;k_{2}\;k_{4}$$

Under the assumption of rapid equilibrium Eq. (A16) reduces to:

(A17) $$\frac{k_{1}}{k_{-1}}=\frac{k_{2}}{k_{-2}}\frac{K_{I}}{K_{\mathrm{II}}}$$

Thus if the translocation rate constants in both directions are assumed to be equal (Eqs. (A11), (A12)), then the substrate dissociation constants on both sides of the membrane also necessarily have to be equal $\left(K_{I}=K_{\mathrm{II}}=K\right)$. Conversely, Eq. (A17) also implies that if the dissociation constants were assumed to be different on both sides, then all translocation rates cannot be equal. Consequently, based on these assumptions, the model as given by Eq. (A7) reduces to:

(A18) $$J_{A}=\frac{2V_{J_{A}}hK\;\left({\left[A\right]}^{I}-{\left[A\right]}^{\mathrm{II}}\right)}{2{\left[A\right]}^{I}{\left[A\right]}^{\mathrm{II}}+K\left(h+1\right)\left({\left[A\right]}^{I}+{\left[A\right]}^{\mathrm{II}}+K\right)}$$

where

$$V_{J_{A}}=\frac{kx_{T}}{2}$$

Here h is dimensionless, K has unit of concentration (µmol l^−1^) and $V_{J_{A}}$ is in µmol m^−2^ min^−1^. The rate of change of the concentration within the vesicle $d{\left[A\right]}^{\mathrm{II}}/dt$ can be obtained by multiplying the flux $J_{A}$ by the area to volume ratio of the vesicle. In this form, Eq. (A18) represents transport of only a single substrate. A similar procedure as described above can be adopted for the transport of multiple substrates. The resulting expression for two substrates is given in Eq. (1).

## Appendix B

In this section the effect of asymmetry in transport parameters is explored further. The schematic of the extended model with explicit translocation rate and dissociation constants is shown in Fig. B1. The corresponding model equation, derived based on the same assumptions as in Eqs. (A1), (A2), (A3), (A4), (A5), (A6), is given in Eq. (B1)

(B1) $$J_{A}=\frac{\left(k_{1}k_{-1}({\left[A\right]}^{I}{\left[B\right]}^{\mathrm{II}}-{\left[A\right]}^{\mathrm{II}}{\left[B\right]}^{I})+k_{-2}k_{1}K_{\mathrm{II}}{\left[A\right]}^{I}-k_{2}k_{-1}K_{I}{\left[A\right]}^{\mathrm{II}}\right)x_{T}}{\left(k_{1}+k_{-1}\right){\left[Tot\right]}^{I}{\left[Tot\right]}^{\mathrm{II}}+\left(k_{-2}+k_{1}\right)K_{\mathrm{II}}{\left[Tot\right]}^{I}+\left(k_{2}+k_{-1}\right)K_{I}{\left[Tot\right]}^{\mathrm{II}}+(k_{2}+k_{-2})K_{\mathrm{II}}\;K_{I}}$$

where

$${\left[Tot\right]}^{i}={\left[A\right]}^{i}+{\left[B\right]}^{i}$$

*Asymmetric bound translocation rate and dissociation constants:* If asymmetry in the translocation rate of the bound transporters $\left(k_{1}\neq k_{-1}\right)$ is assumed, then the other model parameters must be adjusted in accordance with the thermodynamic constraint for a passive process as given in Eq. (A17). If symmetry in the unbound translocation rate constants $\left(k_{2}=k_{-2}=hk\right)$ is assumed, then Eq. (A17) becomes:

(B2) $$\frac{K_{\mathrm{II}}}{K_{I}}=\frac{k_{-1}}{k_{1}}$$

Let $c=K_{\mathrm{II}}/K_{I}$ be the ratio between internal and external dissociation constants, with $K_{\mathrm{II}}=cK$ and $K_{I}=K$, then:

(B3) $$\begin{array}{l} k_{-1}=ck\\ k_{1}=k \end{array}$$

c is then, by definition, the factor that governs the asymmetry in the translocation rates constants. Note that c is unity when symmetry is assumed.

The resulting theoretical predictions for the obligatory exchange model (h=0) and non-obligatory (facilitated) transporter model (for the case h=1) are shown in Fig. B2, using the parameter values of k=1, $x_{T}=1$ and K=0.5 (arbitrary units).

From the results in Fig. B2 it can be observed that a higher reverse translocation rate constant for the bound transporter $\left(k_{-1}\right)$ has minimal effect on the model predictions for tracer uptake (c=10), while a lower uptake rate constant (c=0.1) leads to slower uptake for the cases considered. Notably, the value of c shifts the position of the overshoot in time and affects the peak width, but does not affect the peak height (Fig. B2f).

*Asymmetric unbound translocation rate and dissociation constants:* In addition, asymmetry in the translocation rate constants of the unbound transporter $\left(k_{2}\neq k_{-2}\right)$ was explored, with symmetric bound translocation rate constants $\left(k_{1}=k_{-1}=k\right)$. Eq. (A17) then becomes:

(B4) $$\frac{K_{II}}{K_{I}}=\frac{k_{2}}{k_{-2}}$$

Let $c=K_{II}/K_{I}$ again be the ratio between internal and external dissociation constants, with $K_{II}=cK$ and $K_{I}=K$, then define:

(B5) $$\begin{array}{l} k_{2}=chk\\ k_{-2}=hk \end{array}$$

The resulting theoretical predictions for the obligatory and non-obligatory models are shown in Fig. B3, using the same parameters as before.

For the non-obligatory (facilitative) model, Fig. B3d shows that in absence of internal substrate, a higher forward translocation rate constant for the unbound transporter $\left(k_{2}\right)$ has minimal effect on the model predictions for tracer uptake (c=10), while a lower uptake rate constant (c=0.1) leads to slower uptake. Fig. B3e shows that this effect of c is reversed in the presence of an initial internal substrate concentration equal to the external tracer concentration. In addition, for this situation there is no difference between the obligatory and non-obligatory model (Fig. B3b and e). In contrast, marked differences arise for high initial internal substrate concentrations. The obligatory exchange model in Fig. B3c displays only minor effects, which are due to the difference in dissociation constants depending on c, as unbound translocation rates are zero because h=0. However, for the non-obligatory (facilitative) model in Fig. B3e, a low value of c leads to a higher peak, which decreases more slowly. On the other hand, a high value of c almost completely eliminates the overshoot, since the fast unbound forward translocation rate bypasses tracer uptake in the transport cycle during efflux of initial internal substrate.

*Asymmetric bound and unbound translocation rate, with equal dissociation constants:* Furthermore, the case of asymmetry in the translocation rate constants ($k_{1}\neq k_{-1}$ and $k_{2}\neq k_{-2}$) was explored, with symmetric dissociation constants $\left(K_{I}=K_{\mathrm{II}}=K\right)$, Eq. (A17) then becomes:

(B6) $$\frac{k_{1}}{k_{-1}}=\frac{k_{2}}{k_{-2}}$$

Let $c=k_{1}/k_{-1}$ be the ratio between forward and backward translocation rate constants of the bound transporters with $k_{1}=ck$ and $k_{-1}=k$, then define:

(B7) $$\begin{array}{l} k_{2}=chk\\ k_{-2}=hk \end{array}$$

The resulting theoretical predictions for the obligatory exchange and non-obligatory (facilitated) transport models are shown in Fig. B4, using the same parameters as before.

It can be observed that the results in Fig. B4 are very similar to those in Fig. B2, but note that c has the opposite effect in Eqs. (B3), (B6), acting either on the forward or reverse bound translocation rate constants $k_{1}$ and $k_{-1}$. However, this has the same effect on the overall transport cycle where A is exchanged for B (Fig. B1).

## References

[bib1] Barta E., Drugan A. (2010). Glucose transport from mother to fetus—a theoretical study. J. Theor. Biol..

[bib2] Blumenthal R., Kedem O. (1969). Flux ratio and driving forces in a model of active transport. Biophys. J..

[bib3] Broer S. (2008). Amino acid transport across mammalian intestinal and renal epithelia. Physiol. Rev..

[bib4] Chernyavsky I.L., Jensen O.E., Leach L. (2010). A mathematical model of intervillous blood flow in the human placentone. Placenta.

[bib5] Cleal J.K., Lewis R.M. (2008). The mechanisms and regulation of placental amino acid transport to the human foetus. J. Neuroendocrinol..

[bib6] Cleal J.K., Glazier J.D., Ntani G., Crozier S.R., Day P.E., Harvey N.C., Robinson S.M., Cooper C., Godfrey K.M., Hanson M.A., Lewis R.M. (2011). Facilitated transporters mediate net efflux of amino acids to the fetus across the basal membrane of the placental syncytiotrophoblast. J. Physiol..

[bib7] Devés R., Krupka R.M. (1979). A simple experimental approach to the determination of carrier transport parameters for unlabeled substrate analogs. Biochim. Biophys. Acta (BBA)—Biomembr..

[bib8] Devés R., Krupka R.M. (1979). A general kinetic analysis of transport tests of the carrier model based on predicted relations among experimental parameters. Biochim. Biophys. Acta (BBA)—Biomembr..

[bib9] Fotiadis D., Kanai Y., Palacín M. (2013). The SLC3 and SLC7 families of amino acid transporters. Mol. Aspects Med..

[bib10] Friedman M.H. (2008).

[bib11] Ganapathy M.E., Leibach F.H., Mahesh V.B., Howard J.C., Devoe L.D., Ganapathy V. (1986). Characterization of tryptophan transport in human placental brush-border membrane vesicles. Biochem. J..

[bib122] Ganapathy V., Ganapathy M.E., Tiruppathi C., Miyamoto Y., Mahesh V.B., Leibach F.H. (1988). Sodium-gradient-driven, high-affinity, uphill transport of succinate in human placental brush-border membrane vesicles. Biochem. J..

[bib12] Gill J.S., Salafia C.M., Grebenkov D., Vvedensky D.D. (2011). Modeling oxygen transport in human placental terminal villi. J. Theor. Biol..

[bib13] Glazier J.D., Sibley C.P. (2006). In vitro methods for studying human placental amino acid transport: placental plasma membrane vesicles. Methods Mol. Med..

[bib14] Glazier J.D., Jones C.J.P., Sibley C.P. (1988). Purification and Na+ uptake by human placental microvillus membrane vesicles prepared by three different methods. Biochim. Biophys. Acta (BBA)—Biomembr..

[bib15] Glazier J.D., Cetin I., Perugino G., Ronzoni S., Grey A.M., Mahendran D., Marconi A.M., Pardi G., Sibley C.P. (1997). Association between the activity of the system A amino acid transporter in the microvillous plasma membrane of the human placenta and severity of fetal compromise in intrauterine growth restriction. Pediatr. Res..

[bib16] Harrison L.I., Christensen H.N. (1975). Simulation of differential effects on rates in membrane transport. J. Theor. Biol..

[bib17] Jansson N., Pettersson J., Haafiz A., Ericsson A., Palmberg I., Tranberg M., Ganapathy V., Powell T.L., Jansson T. (2006). Down-regulation of placental transport of amino acids precedes the development of intrauterine growth restriction in rats fed a low protein diet. J. Physiol..

[bib18] Jansson T., Powell T.L. (2006). Human placental transport in altered fetal growth: does the placenta function as a nutrient sensor?—A review. Placenta.

[bib19] Jansson T., Scholtbach V., Powell T.L. (1998). Placental transport of leucine and lysine is reduced in intrauterine growth restriction. Pediatr. Res..

[bib20] Jóźwik M., Teng C., Wilkening R.B., Meschia G., Battaglia F.C. (2004). Reciprocal inhibition of umbilical uptake within groups of amino acids. Am. J. Physiol.—Endocrinol. Metab..

[bib133] Kanai Y., Segawa H., Miyamoto K.-i., Uchino H., Takeda E., Endou H. (1998). Expression Cloning and Characterization of a Transporter for Large Neutral Amino Acids Activated by the Heavy Chain of 4F2 Antigen (CD98). J. Biol. Chem..

[bib21] Khalili-Araghi F., Gumbart J., Wen P.C., Sotomayor M., Tajkhorshid E., Schulten K. (2009). Molecular dynamics simulations of membrane channels and transporters. Curr. Opin. Struct. Biol..

[bib22] Läuger P. (1991).

[bib23] Lewis R.M., Cleal J.K., Hanson M.A. (2012). Review: placenta, evolution and lifelong health. Placenta.

[bib24] Lewis R.M., Glazier J., Greenwood S.L., Bennett E.J., Godfrey K.M., Jackson A.A., Sibley C.P., Cameron I.T., Hanson M.A. (2007). L-serine uptake by human placental microvillous membrane vesicles. Placenta.

[bib25] Lewis R.M., Brooks S., Crocker I.P., Glazier J., Hanson M.A., Johnstone E.D., Panitchob N., Please C.P., Sibley C.P., Widdows K.L., Sengers B.G. (2013). Review: modelling placental amino acid transfer—from transporters to placental function. Placenta.

[bib26] Meier C., Ristic Z., Klauser S., Verrey F. (2002). Activation of system L heterodimeric amino acid exchangers by intracellular substrates. EMBO J..

[bib27] Paolini C.L., Marconi A.M., Ronzoni S., Di Noio M., Fennessey P.V., Pardi G., Battaglia F.C. (2001). Placental transport of leucine, phenylalanine, glycine, and proline in intrauterine growth-restricted pregnancies. J. Clin. Endocrinol. Metab..

[bib28] Parent L., Supplisson S., Loo D.D., Wright E.M. (1992). Electrogenic properties of the cloned Na^+^/glucose cotransporter: II. A transport model under nonrapid equilibrium conditions. J. Membr. Biol..

[bib29] Philipps A.F., Holzman I.R., Teng C., Battaglia F.C. (1978). Tissue concentrations of free amino acids in term human placentas. Am. J. Obstet. Gynecol..

[bib143] Pineda M., Fernández E., Torrents D., Estévez R., López C., Camps M., Lloberas J., Zorzano A., Palacín M. (1999). Identification of a Membrane Protein, LAT-2, That Co-expresses with 4F2 Heavy Chain, an L-type Amino Acid Transport Activity with Broad Specificity for Small and Large Zwitterionic Amino Acids. J. Biol. Chem.

[bib30] Segawa H., Fukasawa Y., Miyamoto K.-i., Takeda E., Endou H., Kanai Y. (1999). Identification and functional characterization of a Na^+^-independent neutral amino acid transporter with broad substrate selectivity. J. Biol. Chem..

[bib31] Sengers B.G., Please C.P., Lewis R.M. (2010). Computational modelling of amino acid transfer interactions in the placenta. Exp. Physiol..

[bib32] Sibley C., Glazier J., D’Souza S. (1997). Placental transporter activity and expression in relation to fetal growth. Exp. Physiol..

[bib33] Sibley C.P., Brownbill P., Dilworth M., Glazier J.D. (2010). Review: adaptation in placental nutrient supply to meet fetal growth demand: implications for programming. Placenta.

[bib34] Staud F., Vackova Z., Pospechova K., Pavek P., Ceckova M., Libra A., Cygalova L., Nachtigal P., Fendrich Z. (2006). Expression and transport activity of breast cancer resistance protein (Bcrp/Abcg2) in dually perfused rat placenta and HRP-1 cell line. J. Pharmacol. Exp. Ther..

[bib35] Stein W.D., Lieb W.R. (1986).

[bib36] Tsitsiou E., Sibley C.P., D’Souza S.W., Catanescu O., Jacobsen D.W., Glazier J.D. (2009). Homocysteine transport by systems L, A and y+L across the microvillous plasma membrane of human placenta. J. Physiol..

[bib37] Turner R.J. (1983). Quantitative studies of cotransport systems: models and vesicles. J. Membr. Biol..

